# Fenofibrate decreased microalbuminuria in the type 2 diabetes patients with hypertriglyceridemia

**DOI:** 10.1186/s12944-020-01254-2

**Published:** 2020-05-23

**Authors:** Xiaomeng Sun, Jia Liu, Guang Wang

**Affiliations:** Department of Endocrinology, Beijing Chao-Yang Hospital, Capital Medical University, 8 Gongren Tiyuchang Nanlu, Chaoyang District, Beijing, 100020 P. R. China

**Keywords:** Type 2 diabetes mellitus, Hypertriglyceridemia, Fenofibrate, Urinary albumin/creatinine ratio

## Abstract

**Background:**

This study was to research the efficacy of fenofibrate in the treatment of microalbuminuria in the patients with type 2 diabetes mellitus (T2DM) and hypertriglyceridemia.

**Methods:**

Type 2 diabetic patients (56) with microalbuminuria and hypertriglyceridemia aged 30 to 75 were randomly divided into the fenofibrate treatment group(*n* = 28) and the control group (n = 28) for 180 days. Urinary microalbumin /creatinine ratio (UACR) and other metabolic parameters were compared at baseline, during treatment and after treatment.

**Results:**

After 180 days, the reduction of level of fasting blood glucose (FBG) and glycosylated hemoglobin (HbA1c) between two groups showed no difference. In the treatment group, uric acid (UA) (296.42 ± 56.41 vs 372.46 ± 72.78), triglyceride (TG) [1.51(1.17, 2.06) vs 3.04(2.21, 3.29)], and UACR [36.45 (15.78,102.41) vs 129.00 (53.00, 226.25)] were significantly decreased compared with the baseline. The high-density lipoprotein cholesterol (HDL-C) levels were significantly increased (1.22 ± 0.26 vs 1.09 ± 0.24) compared with the baseline. The decrease in UACR [− 44.05(− 179.47, − 12.16) vs − 8.15(− 59.69, 41.94)]in treatment group was significantly higher compared with the control group. The decrease in UACR was positively associated with the decreases in TG (*r* = 0.447, *P* = 0.042) and UA (*r* = 0.478, *P* = 0.024) after fenofibrate treatment.

**Conclusion:**

In the patients with hypertriglyceridemia and type 2 diabetes mellitus, fenofibrate can improve microalbuminuria and do not increase the deterioration of glomerular filtration rate.

**Trial registration:**

ClinicalTrials.gov identifier: NCT02314533, 2014.12.9

## Background

Diabetic nephropathy (DN) has been considered as one of the important microvascular complication of diabetes mellitus, if left untreated, it can lead to kidney failure and renal dialysis or kidney transplantation [1]. Hypoglycemic, hypotensive, and lipid lowering drugs are the main treatment options for DN at present. However, there are still some patients who continue to experience DN progression even after intensive hypoglycemic therapy, statin treatment, and blood pressure reaching the standard levels. Our previous studies have shown that fenofibrate therapy can significantly reduce insulin resistance and the secretory load of β cells [2]. Fenofibrate could improve plasma level of tetrahydrobiopterin (BH4) and protect endothelial function [3, 4]. The Fenofibrate Intervention and Event Lowering in Diabetes (FIELD) study suggested that treatment of fenofibrate can reduce albuminuria and prevent the progression of diabetic nephropathy [5, 6]. The Action to Control Cardiovascular Risk in Diabetes (ACCORD) study suggested that a lower incidence of both microalbuminuria and macroalbuminuria was noted in the fenofibrate group [7]. However, all these studies are long-term studies with observation periods of 3–5 years. It is unclear whether fenofibrate could impact microalbuminuria on the early stage of the treatment. Moreover, urinary microalbumin/creatinine ratio (UACR) is less affected by diet and urine concentration. We designed this study to evaluate fenofibrate’s effect on microalbuminuria change and estimated glomerular filtration rate (eGFR) in Chinese type 2 diabetes patients with hypertriglyceridemia.

## Methods

### Study design and participants

This is a randomized (no placebo) controlled study to evaluate the efficacy of fenofibrate on microalbuminuria in type 2 diabetes mellitus (T2DM) patients with hypertriglyceridemia. The study enrolled 56 subjects to meet scientific and regulatory objectives without enrolling an undue number of subjects in alignment with ethical considerations. A total of 56 T2DM patients were enrolled by the endocrinology department at Beijing Chao-Yang Hospital during February 2015 and July 2018. The diagnosis of T2DM was in accordance with the World Health Organization (WHO) criteria of 2013 [8]. All the patients with HbA1c levels < 8% and with microalbuminuria were eligible for the study. Diagnosis of microalbuminuria was determined by two morning spot urine samples on different days, defined as UACR between 30 and 300 mg/g. All patients had been treated with statin monotherapy at low to moderate doses at least 2 months prior to enrollment and planned to continue the same type and dose of statin, but their triglyceride (TG) levels were still greater than 1.7 mmol/L and lower than 5.6 mmol/L. Additionally, the blood pressure levels of all the patients should lower than 140/90 mmHg. The 56 patients were randomly divided to the fenofibrate treatment group (group A) or control group (group B) according to the random number table after subject enrollment condition was confirmed. Fenofibrate (Lipanthyl®) 200 mg capsule was administered orally with breakfast once daily according to the Chinese prescription information of Lipanthyl®. During treatment, the hypoglycemic and hypotensive drugs were no longer adjusted. Hypoglycemic drugs included metformin, α-glucosidase inhibitors, sulfonylureas, glinides, and insulin. The main lipid-lowering drugs were atorvastatin, rosuvastatin, simvastatin, and pravastatin. The exclusion criteria were pregnancy or possible pregnancy, cardiovascular disease (CVD), hepatic insufficiency (ALT or AST > 2*ULN), renal insufficiency [estimate glomerular filtration rate (eGFR) < 60 ml/min estimated from Modification of Diet in Renal Disease (MDRD) equation], thyroid disease, infectious disease, cancer, and/or systemic inflammatory diseases. The study received approval from the Ethics Committee of Beijing Chao-yang Hospital. All participants gave their written, informed consent to participate. This study was registered with ClinicalTrials.gov. (ClinicalTrials.gov identifier: NCT02314533). The study was in compliance with the content of the Declaration of Helsinki. All participants were required to attend 3 study visits: visit 1, the screening visit; visit 2, fenofibrate treatment for 90 days; visit 3, fenofibrate treatment for 180 days. Fasting blood samples and urine sample were collected at every visit. All subjects underwent clinical assessments of age, sex, height, and weight at the first visit. Age, and weight were assessed at every visit. The total cholesterol (TC), high-density lipoprotein cholesterol (HDL-C), TG, low-density lipoprotein cholesterol (LDL-C), serum creatinine (Scr), urinary creatinine, and uric acid (UA) were enzymatically determined (Siemens Advia 2400, Siemens Healthcare Diagnostics Inc., Tarrytown, New York, USA). Urinary albumin was measured using immunoturbidimetric assay (Siemens Advia 2400, Siemens Healthcare Diagnostics Inc., Tarrytown, New York, USA). Fasting serum insulin (FINS) was measured using the chemiluminescence immunoassay (System Centaur XP, reference interval:1.9–23 mIU/mL; Siemens Healthcare Diagnostics, Inc., Tarrytown, New York, USA). HbA1c was determined by high-performance liquid chromatography (HLC-723G7 analyzer, reference interval: 4–6%; Tosoh Corporation, Tokyo, Japan). Body mass index (BMI) was calculated as weight (kg)/height^2^ (m^2^). The homeostasis model assessment index of insulin resistance (HOMA-IR) was calculated as follows: HOMA-IR = fasting insulin (uU/mL) × fasting plasma glucose (mmol/L)/ 22.5; The homeostasis model assessment for β-cell function(HOMA-β) was calculated as follows: HOMA-β = 20 × fasting insulin(uU/mL)/(fasting plasma glucose(mmol/L)-3.5) × 100%; UACR = urinary microalbumin (mg/dL) × 1000 / urinary creatinine (umol/L) × 1000/113.1 × 1000/100 (mg/g); eGFR estimated using the MDRD equation: male: eGFR = 186 × Cr(mg/dl)-1.154 × (age)-0.203 (ml/min/1.73m^2^); female: eGFR =186 × Cr(mg/dl)-1.154 × (age)-0.203 × 0.742 (ml/min/1.73m^2^).

## Statistical analyses

Data were analyzed with SPSS version 19.0 (IBM Corp., Armonk, NY, USA). Continuous data, such as, BMI, age, systolic blood pressure (SBP), diastolic blood pressure (DBP), fasting blood glucose (FBG), HbA1C, TC, HDL-C, LDL-C, UA, and Scr were expressed as mean ± standard deviation. Because some data, such as TG, FINS, HOMA-IR, HOMA-β, eGFR, and UACR were not normally distributed, the values are provided as medians (interquartile range, IQR). Normally distributed data were analyzed by t-test. Logarithmically transformed values were used in the statistical analyses. Two-tails paired t-test was used in parameters from the baseline values within group. Independent sample t-test was used to compare differences between groups at baseline and after treatment. The correlation of the changes in UACR and other parameters were analyzed using the Spearman correlation. The differences of proportions were analyzed by chi-square test. All statistical tests are two-tailed, with *P*-value < 0.05 considered to be statistically significant.

## Results

### Patients’ baseline characteristics

The baseline clinical characteristics are summarized in Table 1. No differences were found in age, sex, BMI, SBP, DBP, FBG, HbA1c, TC, TG, HDL-C, LDL-C, UA, Scr, FINS, HOMA-IR, HOMA-β, eGFR, UACR, or the use of antihypertensive drugs, cholesterol-lowering drugs, and hypoglycemic drugs in two groups (Table 1).

**Table 1:** Clinical characteristics at baseline parameters and of all subjects

Group	Control group (*n* = 28)	Fenofibrate group (*n* = 28)	*P*
Sex (male/%)	17/60.7	20/71.4	0.604
Age (year)	56.03±12.32	54.64±10.09	0.593
Duration of diabetes (years)	5.21±2.02	5.83±1.39	0.716
Hypertension (number/%)	10/35.7	11/39.3	0.500
AECI/ARB (number/%)	7/25.0	8/28.6	0.768
CCB (number/%)	5/17.9	5/17.9	0.636
Metformin (number/%)	15, 53.6	13, 46.4	0.601
α-Glucosidase inhibitors (number/%)	20, 71.42	22, 78.6	0.546
Sulfonylureas (number/%)	12, 42.9	11, 39.3	0.791
Glinides (number/%)	10, 35.7	11, 39.3	0.787
Insulin (number/%)	6, 21.4	8, 28.6	0.786
Insulin/Oral medications (%)	22.2	28.6	0.372
Simvastatin (number/%)	12/42.9	10/35.7	0.392
Atorvastatin (number/%)	9/28.0	10/35.7	0.591
Rosuvastatin (number/%)	3/10.7	2/7.1	0.500
pravastatin(number/%)	4/14.3	6/21.4	0.364
SBP (mmHg)	137.75±13.77	137.71±9.59	0.996
DBP (mmHg)	75.00±5.60	76.29±7.87	0.782
BMI, kg/m^2^	27.28±3.99	28.32±4.36	0.429
FPG, mmol/L	7.92±2.16	8.27±1.31	0.606
HbA1c, %	7.62±0.87	7.48±0.86	0.480
TG, mmol/L	2.97(2.13,3,31)	3.04(2.21, 3.29)	0.606
TC, mmol/L	4.59±1.14	4.79±1.01	0.474
LDL-C, mmol/L	2.47±0.90	2.55±0.94	0.670
HDL-C, mmol/L	1.02±0.20	1.09±0.24	0.275
Scr, μmol/L	69.56±15.43	65.46±20.98	0.349
UA, μmol/L	388.44±126.99	372.46 ± 72.78	0.803
UACR, mg/g	105.00(64.00, 165.00)	129.00 (53.00, 226.25)	0.095
eGFR ml/min/1.73m^2^	94.01 (80.95, 120.95)	105.36 (99.68, 116.92)	0.066
FINS, μIU/mL	12.9 (6.75, 15.78)	12.05 (8.35, 14.95)	0.101
HOMA-IR	4.50 (3.13, 5.95)	4.27 (3.05, 5.35)	0.105
HOMA-β	42.04 (25.33,75.23)	49.51 [35.85, 70.05]	0.127

Data are means ± SD or medians (interquartile range) or n (%)

*AECI/ARB* Angiotensin Converting Enzyme Inhibitors/Angiotensin receptor antagonist, *CCB* Ca-Antagonists, *SBP* systolic blood pressure, *DBP* diastolic blood pressure, *BMI* body mass index, *FBG* fasting blood glucose, *TG* triglycerides, *TC* total cholesterol, *LDL-C* low-density lipoprotein cholesterol, *HDL-C* high-density lipoprotein cholesterol, *Scr* serum creatinine, *UA* uric acid, *UACR* urinary albumin creatinine ratio, *eGFR* estimated glomerular filtration rate, *FINS* fasting serum insulin, *HOMA-IR* homeostasis model assessment for insulin resistance, *HOMA-β* homeostasis model assessment for β-cell function

### Changes in metabolic parameters after fenofibrate treatment

Compared with the baseline, FBG and HbA1c all significantly decreased in the treatment group and the control group at 90 days and 180 days. There was no difference between the two groups in FBG and HbA1c at 90 days and 180 days.

In the treatment group, after 90 days of fenofibrate treatment, we found that the levels of UA (290.42 ± 76.76 vs 372.46 ± 72.78), and TG [1.71 (1.27, 2.31) vs 3.04(2.21, 3.29)] were significantly lower than the baseline. After 180 days of fenofibrate treatment, the levels of UA (296.42 ± 56.41 vs 372.46 ± 72.78), TG [1.51 (1.17, 2.06) vs 3.04(2.21, 3.29)], UACR [36.45 (15.78,102.41) vs 129.00 (53.00, 226.25)], and HOMA-IR [2.77(1.98, 3.44) vs 4.27(3.05, 5.35)] were significantly lower at 180 days than at baseline, while HDL-C (1.22 ± 0.26 vs 1.09 ± 0.24) was significantly higher at 180 days than at baseline(all *P* < 0.05). No differences were found in BMI, TC, LDL-C, Scr, HOMA-β, and eGFR among the three visits.

In the control group, HOMA-IR [3.12(2.01, 3.87) vs 4.50(3.13, 5.95)] were significantly lower at 180 days than at baseline. There were no differences in BMI, TC, TG, HDL-C, LDL-C, UA, Scr, FINS, HOMA-β, eGFR, and UACR among the three visits.

The decreases in UA [− 92.5(− 145, − 21) vs 0.00(− 60.00,45.00)], TG [− 1.27(− 1.77, − 0.24) vs-0.64(− 0.96,0.42)] and eGFR [− 10.70(− 22.78, − 8.31) vs − 2.27(− 7.33,4.63)]in the treatment group showed greater decline compared the control group at 90 days. The decreases in UACR [− 44.05(− 179.47, − 12.16) vs − 8.15(− 59.69, 41.94)], UA [− 66(− 111.00, − 34.00) vs − 16.00(− 43.75, − 16.00)] and TG [− 1.91(− 1.12, − 0.53) vs-0.22(− 1.21,0.19)] showed greater decline compared the control group at 180 days. The increase in Scr was significantly higher at 90 days in the treatment group than the control group. The increase in HDL-C was significantly higher at 180 days in treatment group than the control group. There was no difference between the two groups in the increase of Scr and the decrease of eGFR at 180 days. (Table 2).

**Table 2:** The changes of Clinical characteristics after 90 and 180 days follow up

Group	Control (*n* = 28)	Fenofibrate treatment (*n* = 28)
SBP (mmHg)		
90 days	-2.00(-7.00,3.00)	-1.00(-3.00, -1.00)
180 days	-2.50(-8.00, 5.00)	-1.00(-7.00, 4.00)
DBP ( mmHg)		
90 days	-0.80(-3.00, 4.00)	-1.00(-3.00, -1.00)
180 days	-1.00(-2.00, 5.00)	-1.20(-2.00, 0.25)
BMI, kg/m^2^		
90 days	-0.26(-1.56,0.12)	-0.20(-1.16,0.15)
180 days	-0.22(-1.32,0.17)	-0.21(-1.01,0.22)
FPG, mmol/L		
90 days	-0.43(-1.70,0.50)	-0.37(-1.77, 0.32)
180 days	-0.24(-2.44, 0.39)	-0.68(-2.81,0.19)
HbA1c, %		
90 days	-0.60(-1.30,0.10)	-0.40(-1.35, -0.10)
180 days	-0.90(-1.40, -0.25)	-0.70(-1.38, -0.10)
TG, mmol/L		
90 days	-0.64(-0.96,0.42)	-1.27(-1.77, -0.24) *
180 days	-0.22(-1.21,0.19)	-1.91(-1.12, -0.53) ^**#**^
TC, mmol/L		
90 days	-0.19(-1.26,0.11)	-0.19(-0.77, -0.19)
180 days	0.22(-0.29, 0.53)	-0.21 (-0.87,0.31)
LDL-C, mmol/L		
90 days	-0.10(-0.80,0.10)	-0.15(-0.50, 0.72)
180 days	0.25(-0.18, 0.40)	-0.20(-0.40,0.40)
HDL-C, mmol/L		
90 days	0.00(0.00,0.01)	0.20(0.10,0.20) *
180 days	0.00(0.00,0.01)	0.16(0.00,0.22) ^**#**^
Scr, μmol/L		
90 days	1.70(-2.40,5.20)	7.60(3.80, 12.40) *
180 days	4.35(-0.95, 9.80)	4.25 (-1.75, 15.60)
UA, μmol/L		
90 days	0.00(-60.00,45.00)	-92.5(-145, -21) *
180 days	-16.00(-43.75, -16.00)	-66(-111.00, -34.00) ^**#**^
UACR, mg/g		
90 days	-20.87(-61.16,15.72)	-41.39(-103.81, -1.44)
180 days	-8.15(-59.69, 41.94)	-44.05(-179.47, -12.16) ^**#**^
eGFR ml/min/1.73m^2^		
90 days	-2.27(-7.33,4.63)	-9.25(-22.10, -8.28) *
180 days	-5.63(-12.10,1.12)	-7.26(-21.34, -1.37)
FINS, μIU/mL		
90 days	-0.60(-3.45,2.12)	-0.90(-4.50,2.10)
180 days	-0.25(-5.15, 2.35)	-1.40(-6.90, 1.81)
HOMA-IR		
90 days	-0.35(-1.98,0.64)	-0.25(-2.77, 0.45)
180 days	-1.09(-2.68, -1.09)	-0.94(-3.15, 0.58)
HOMA-β		
90 days	6.19(-38.88, 20.64)	3.62(-6.21,25.04)
180 days	6.98(-39.58, 34.25)	4.75(-21.91, 30.38)

Data are means ± SD or medians (interquartile range) or n (%)

*AECI/ARB* Angiotensin Converting Enzyme Inhibitors/Angiotensin receptor antagonist, *SBP* systolic blood pressure, *DBP* diastolic blood pressure, *BMI* body mass index, *FBG* fasting blood glucose, *TG* triglycerides, *TC* total cholesterol, *LDL-C* low-density lipoprotein cholesterol, *HDL-C* high-density lipoprotein cholesterol, *Scr* serum creatinine, *UA* uric acid, *UACR* urinary albumin to creatinine ratio, *eGFR* estimated glomerular filtration rate, *FINS* fasting serum insulin, *HOMA-IR* homeostasis model assessment for insulin resistance, *HOMA-β* homeostasis model assessment for β-cell function

*P<0.05 vs. the 90 days of control group. ^#^*P*<0.05 vs. the 180 days of control group

### Correlations between the decrease in UACR and changes in other variables after 180 days fenofibrate treatment

**Fig. 1:**
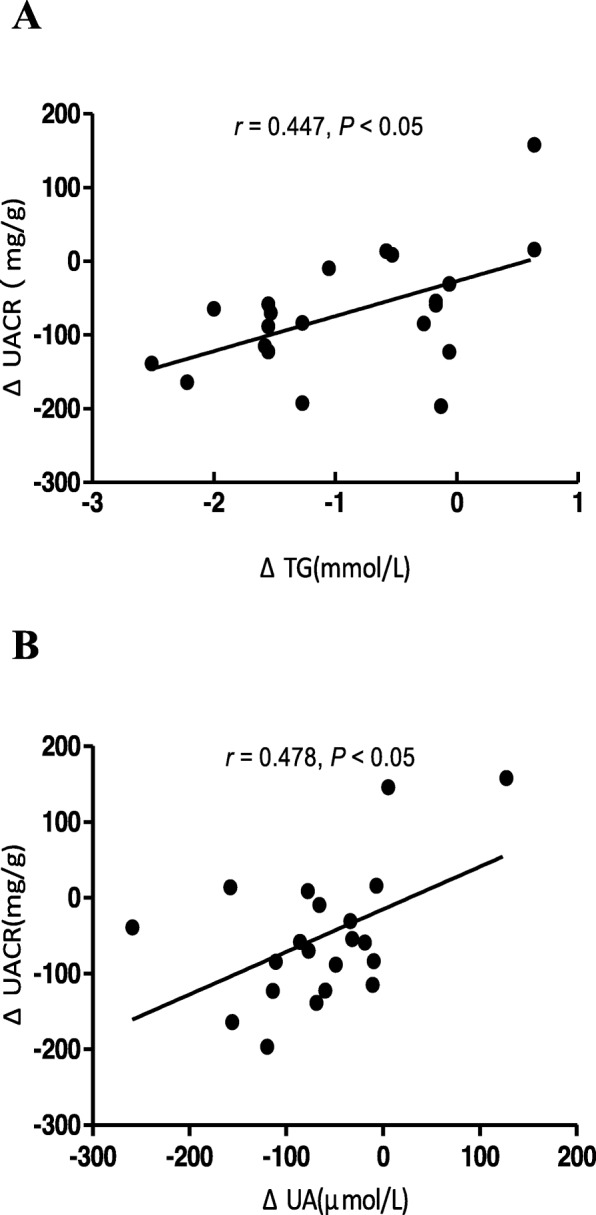
Correlations between the decrease in UACR and changes of TG and UA after fenofibrate treatment

In the fenofibrate group, the decrease in UACR (ΔUACR) was positively associated with the decreases in TG(ΔTG) (*r* = 0.447, *P* = 0.042) and UA(ΔUA) (*r* = 0.478, *P* = 0.024) after fenofibrate treatment (Fig. 1). In our study, we found no significant relationship between the decrease in UACR and the change of age, BMI, TC, HDL-C, LDL-C, Scr, FINS, HOMA-IR, or eGFR.

## Discussion

In this study, at 180 days, compared with the control group, the levels of UACR, UA, and TG were significantly decreased while the levels of HDL-C were significantly increased. The decreases in UACR, UA and TG showed greater decline compared the control group at 180 days. Correlation analysis suggested that the decrease in UACR was positively associated with the decrease in TG and UA after fenofibrate treatment. We found that on the basis of the treatment of blood glucose, blood pressure, and lipids, the treatment of controlling TG could still further reduce UACR.

DN is a very important diabetic microvascular complication. If left untreated, it can lead to hemodialysis and renal transplantation [1]. However, DN can be reversed if diagnosed and treated it on the early stage. Glomerulosclerosis and tubular necrosis, thickening of the basement membranes of the glomeruli and tubules, and dilation of mesangial cells all contribute to the development of DN. These changes can lead to proteinuria, the level of serum creatinine increased, and eventually to decreased glomerular filtration rate [9]. Diabetes and hyperlipidemia cause renal lipid accumulation. At the same time, lipid toxicity due to accumulation of lipids in the mesangium may accelerate the progression of DN [10]. Several studies have shown that peroxisome proliferator-activated receptor α (PPARα) agonist could inhibit renal inflammation and fibrosis and prevent renal oxidative stress [11, 12]. Our previous studies have shown that fenofibrate therapy can significantly reduce insulin resistance and the secretory load of β cells [2]. Fenofibrate could improve plasma levels of tetrahydrobiopterin (BH4) by increasing the guanosine 5-triphosphate cyclohydrolase-I expression and protect endothelial function [3, 4]. Several studies have shown that BH4 can improve endothelial function in patients with diabetes and hypercholesterolemia [12–14]. A study by Xu et al. [15] showed that PPARα or AMP-activated protein kinase α (AMPKα) inhibitors can reverse vasodilation of the aorta. Treatment of fenofibrate can increase the expression of PPAR and induce liver kinase B1 (LKB1) translocation and activation of AMPK, thus activating nitric oxide synthase 3 (eNOS), improving endothelium-dependent dilation of vessels, increasing nitric oxide (NO) levels, and decreasing the role of renal injury markers and the vasoconstrictor prostaglandin. Several animal studies have shown [16, 17] that fenofibrate treatment of db/db mice can not only reduce the expansion of mesangial matrix and glomerular hypertrophy, but also reduce collagen deposition and the expression of transforming growth factor-1 in renal tissue, thus significantly reducing proteinuria and glomerular fibrosis. The Diabetes Atherosclerosis Intervention Study (DAIS) showed that worsening of albumin excretion was reduced after the fenofibrate treatment [18]. The FIELD study suggested that treatment of fenofibrate can reduce albuminuria and prevent the progression of DN [5, 6]. The ACCORD study suggested that [7] a lower incidence of both micro-albuminuria and macro-albuminuria was noted in the fenofibrate group. A small sample size study showed that gemfibrozil might mitigate the progression of microalbuminuria in non-insulin-dependent diabetic patients [19]. A study by Frazier et al. [20] showed that there is lower incidence of microalbuminuria after treatment with fenofibrate for 4 years. These results suggest that the use of fenofibrate may improve lipid toxicity induced by renal lipid accumulation, resulting in decreased urinary microalbumin. We noticed that the increase in Scr and the decrease in eGFR in the treatment group was greater than that in the control group at the 90 days. There was no difference between the two groups in increase in Scr and decrease in eGFR at 180 days. This result was similar to the previous experiment [21–23]. Although there was no good explanation for this phenomenon, we thought the GFR was higher in the early stages of diabetic nephropathy, as the blood sugar and blood lipid decreased, the high filtration state was improved in the short term, so in the treatment group, the rise of creatinine and the decline of GFR were significantly more obvious.

Our study showed that UA levels were significantly lower after fenofibrate treatment. Correlation analysis showed that the decrease in UACR was positively associated with the decrease in UA after fenofibrate treatment. Jung [24] et al. study show that fenofibrate have an additionally role of reducing UA levels in patients having high TG levels. Several [25–28] studies have shown that fenofibrate can reduce UA levels in patients, especially in those patients with coexisting hyperlipidemia. It is believed that fenofibrate can reduce serum UA levels by increasing UA excretion. Several studies have reported [29] that fenofibrate could inhibit the renal organic anion transporter urate transporter 1 and increase urinary excretion of UA to decrease serum UA levels. Several studies [30–32] have shown that serum UA and microalbuminuria are significantly positively associated in T2DM patients.

### Limitations

Our study had several limitations. The present study is small and single-center, and the results may be biased; thus, it requires further confirmation by large-scale, multi-center clinical studies. Moreover, it would be better if there were cell-based and animal studies to demonstrate our result.

## Conclusions

Fenofibrate could reduce progression to microalbuminuria and does not increase eGFR impairment in T2DM patients with hypertriglyceridemia.

## Data Availability

The datasets generated and/or analysed during the current study are not publicly available due individual privacy but are available from the corresponding author on reasonable request.

## Abbreviations

T2DM: Type 2 diabetes mellitus
UACR: Urinary microalbumin /creatinine ratio
FBG: Fasting blood glucose
HbA1c: Glycosylated hemoglobin
UA: Uric acid
TG: Triglyceride
HDL-C: High-density lipoprotein cholesterol
DN: Diabetic nephropathy
FIELD: The fenofibrate intervention and event lowering in diabetes
ACCORD: The action to control cardiovascular risk in diabetes
eGFR: estimated glomerular filtration rate
WHO: World Health Organization
CVD: Cardiovascular disease
MDRD: Modification of diet in renal disease
TC: Total cholesterol
LDL-C: Low-density lipoprotein cholesterol
Scr: Serum creatinine
UA: Uric acid
BMI: Body mass index
HOMA-IR: Homeostasis model assessment index of insulin resistance
HOMA-β: Homeostasis model assessment for β-cell function
SBP: Systolic blood pressure
DBP: Diastolic blood pressure
PPARα: Peroxisome proliferator-activated receptor α
BH4: Tetrahydrobiopterin
AMPKα: AMP-activated protein kinase α
LKB1: Liver kinase B1
eNOS: Activating nitric oxide synthase 3
NO: Nitric oxide
DAIS: The diabetes atherosclerosis intervention study
